# The KT Jeang Retrovirology prize 2018: Eric Freed

**DOI:** 10.1186/s12977-018-0430-5

**Published:** 2018-07-02

**Authors:** Retrovirology Editorial

**Affiliations:** London, UK

**Fig. 1:**
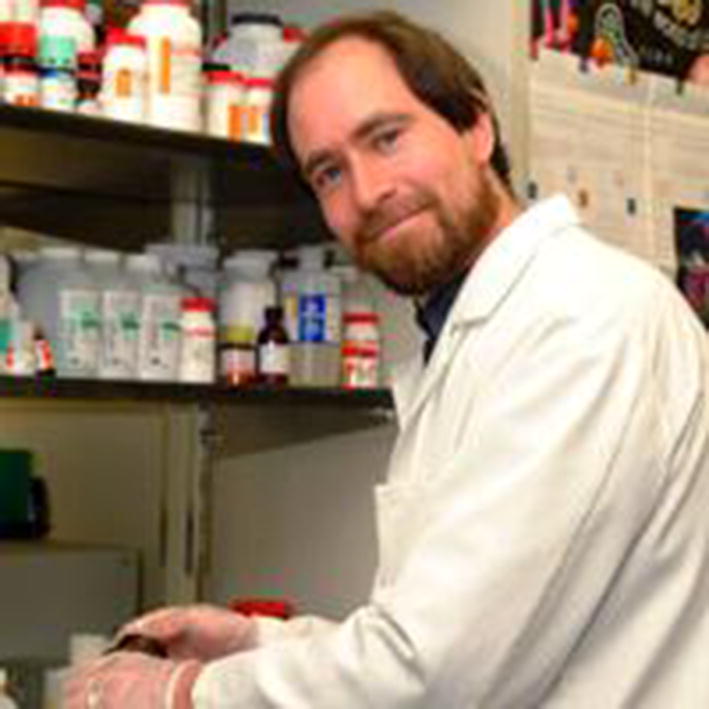
Eric Freed

Dr. Eric Freed did his undergraduate studies at Penn State University, where he had the good fortune of becoming involved in research on the *src* oncogene in the laboratory of Dr. David Shalloway [1]. This early research experience sparked a long-standing interest in viruses—and, more specifically—retroviruses (Fig. 1).

In 1985, Freed began his Ph.D. studies at the University of Wisconsin, Madison, in the laboratory of Dr. Rex Risser. Although research in the Risser laboratory was primarily focused on cell transformation by Abelson murine leukemia virus (MLV), Freed decided from the outset of his Ph.D. research to pursue a different direction—studying membrane fusion mediated by retroviral envelope (Env) glycoproteins. He began by defining the sequence required for MLV Env precursor processing, which he showed was essential for Env-mediated fusion and viral infectivity [2]. In 1988, Freed’s research began to shift from MLV to HIV-1. Freed noted sequence similarities between the MLV and HIV-1 Env glycoprotein precursor cleavage sites, an observation that led him to characterize the HIV-1 Env cleavage sequence [3], and to define the function of the hydrophobic sequence—the gp41 fusion peptide—located immediately downstream of the Env precursor cleavage site [4]. This early work also identified the V3 loop of HIV-1 gp120 (known at that time as the principal neutralizing determinant) as a region of Env critical for membrane fusion [5]. The observation that a gp41 fusion peptide mutation elicited a strong dominant-negative effect on membrane fusion led Freed to speculate that Env likely functions as a multimer [6]. Tragically, Freed’s mentor Rex Risser passed away in 1990, at age 42, just months before Dr. Freed’s Ph.D. thesis defense. Dr. Freed finished his Ph.D. research, and did a 1-year postdoctoral fellowship, under the mentorship of Nobel laureate Dr. Howard Temin; Freed’s work during this time continued to focus on elucidating the function of HIV-1 and HIV-2 Env glycoproteins [7, 8].

In 1992, Freed moved to the NIAID/NIH in Bethesda, Maryland to continue postdoctoral research with Dr. Malcolm Martin. Although Freed maintained his focus on HIV-1, his interests shifted to Gag and virus assembly. During this time, much of his research centered on the matrix (MA) domain of Gag and its role in Gag trafficking, membrane binding, Env incorporation, and virus entry [9–16]. Using oligonucleotide site-directed mutagenesis with M13-derived phage vectors, Freed introduced a large number of mutations into the MA domain of HIV-1 Gag. Two classes of mutations turned out to be particularly interesting: those that blocked Env incorporation into virions, and those, located in a highly basic region of MA, that retargeted virus assembly from the plasma membrane to an intracellular compartment [9–11, 14–16]. His research also addressed the then-controversial hypothesis that MA regulates HIV-1 nuclear import at an early step post-infection [17–19].

In 1995, Freed published a study demonstrating that the virus budding activity of the p6 domain of Gag, originally described by Göttlinger et al. [20], maps to a Pro-Thr-Ala-Pro (PTAP) motif [21]. Within a several-month period from late 2001 to early 2002, four groups (those of Carol Carter, Wesley Sundquist, Paul Bieniasz, and Freed) showed that the PTAP motif of p6 promotes virus budding by binding directly to the ESCRT-I component Tsg101 [22–25]. It soon became clear that PTAP motifs play central roles in protein–protein interactions required for ESCRT activity (e.g., in multivesicular body biogenesis and cytokinesis), adding to the long list of instances in which research in virology has provided fundamental insights into our understanding of molecular and cell biology.

Freed became a tenure-track investigator in 1997 and received tenure from the NIAID and NIH 5 years later (2002). The following year, in 2003, Freed moved his laboratory to the HIV Drug Resistance Program (HIV DRP) at the National Cancer Institute in Frederick, Maryland. The HIV DRP had been founded by John Coffin several years earlier.

Freed’s research in the early 2000s continued to address host cell determinants involved in virus assembly and Env incorporation into virus particles. Freed’s postdoctoral fellow Akira Ono demonstrated that HIV-1 assembly takes place in lipid rafts at the plasma membrane [26], and that targeting of Gag to the plasma membrane relies on the lipid phosphatidylinositol-4,5-bisphosphate [PI(4,5)P_2_] [27]. Ono and Freed showed that depletion of PI(4,5)P_2_ from the plasma membrane of virus-producing cells produced the same phenotype that Freed had earlier described for MA basic residue mutations—the retargeting of Gag to an intracellular compartment—establishing PI(4,5)P_2_ as a key cellular determinant in the regulation of Gag trafficking to the plasma membrane. Others, most notably Jamil Saad and Michael Summers, subsequently showed that the basic region of MA interacts directly with PI(4,5)P_2_ [28].

A theme in Freed’s research for many years has been the interplay between the gp41 cytoplasmic tail and the MA domain of Gag. His early work had shown that single amino acid mutations in MA block Env incorporation, and that this block can be overcome by deleting the gp41 cytoplasmic tail [9]. Freed’s postdoctoral fellow Tsutomu Murakami showed that the long cytoplasmic tail of gp41 is required for Env incorporation and virus replication in physiologically relevant cell types (e.g., most T cell lines and primary T cells) but is not required in adherent cell lines like 293T and HeLa, or in the MT-4 T-cell line [9]. These results suggested that cellular factor(s) promote Env incorporation in a gp41 cytoplasmic tail-dependent manner, a hypothesis that continues to be an active area of investigation. Murakami also showed that defects in Env incorporation and virus replication induced by a small deletion in the gp41 cytoplasmic tail could be rescued by a point mutation in MA, consistent with cross-talk between MA and gp41 [29]. In vitro studies by other groups [30–32] had shown that MA is capable of forming trimers; recently, Freed’s postdoctoral fellow Philip Tedbury showed that a mutation at the putative trimer interface can globally rescue Env incorporation defects [33] and provided direct evidence, by using a cross-linking approach, that MA is indeed trimeric in HIV-1 virions and that MA trimerization is critical to Env incorporation [34]. Work during this period also focused on host cell proteins and pathways involved in HIV-1 Gag targeting and virus budding [35–40].

An additional focus of Freed’s research since the early 2000s has been the development of HIV-1 maturation inhibitors [41–45]. In collaboration with researchers at Panacos Pharmaceuticals, including Feng Li and Carl Wild, Freed’s group showed in 2003 that the first-in-class maturation inhibitor dimethylsuccinyl betulinic acid (PA-457 or bevirimat) blocks virus maturation by preventing the processing of the capsid-spacer peptide 1 (CA-SP1) Gag cleavage intermediate to mature CA [41], an observation made independently shortly thereafter by Chris Aiken’s laboratory [46]. Bevirimat displayed antiviral activity in phase IIb clinical trials [47]; although its activity was compromised by polymorphisms in SP1 [43, 48], this work established proof-of-concept that using a small molecule to block an individual Gag cleavage site can be an effective antiviral strategy in humans. Significant insights into the maturation inhibitor target and mechanism of action were gained from Freed’s work on a structurally distinct maturation inhibitor, known as PF-46396, originally reported by Pfizer [44]. In collaboration with DFH Pharma (headed by Carl Wild) and Hetero Drugs, recent work on maturation inhibitors in the Freed lab has been focused on developing highly potent and broadly active maturation inhibitors that overcome the loss of activity imposed by sequence polymorphisms in SP1. Parallel basic research in Freed’s lab, greatly assisted by cryo-electron tomography and solid-state NMR with collaborators Alasdair Steven and Tatyana Polenova [49–52], and independently by other structural biologists (e.g., Owen Pornillos and John Briggs [53, 54]), has suggested that maturation inhibitors block CA-SP1 processing by stabilizing a highly dynamic, six-helix bundle that forms at the CA-SP1 junction. This work, which relied heavily on the identification and characterization of compound-resistant viruses, is intellectually satisfying in that it illustrates how translational studies on antiviral drug candidates can provide insights into basic virology and structural biology. The maturation inhibitor research in the Freed lab has been carried out by a series of talented postdoctoral fellows (Ritu Gaur, Catherine Adamson, Kayoko Waki, Emiko Urano, and Mariia Novikova), a technician (Sherimay Ablan), and postbaccalaureate interns (Rebecca Mandt, Nishani Kuruppu, Justin Kaplan, Phuong Pham, and Hannah Carter). Other projects currently ongoing in the Freed laboratory involve identifying host cell machinery involved in Env trafficking and incorporation (postdoc Melissa Fernandez); late-acting cellular inhibitory factors that target virus assembly and Env biogenesis, stability and trafficking (postdocs James Kirui and Cheng Man Lun and staff scientist Abdul Waheed); and characterizing a panel of Env mutants that confer resistance to antiretrovirals (postdoc Rachel Van Duyne).

Research in the Freed laboratory has benefited greatly from a number of productive and rewarding collaborations with investigators both within and outside of the NIH, including Schuyler van Engelenburg, Jennifer Lippincott-Schwartz, Juan Bonifacino, James Hurley, Michael Summers, Judith Levin, Eric Barklis, Asim Debnath, Simon Cocklin, Shan-Lu Liu, and Sriram Subramaniam, to name a few [36, 39, 55–74].

In 2014, Freed became Deputy Director of the HIV DRP (under then-Director Stephen Hughes) and in 2015 became Director of the HIV DRP, which was renamed the HIV Dynamics and Replication Program. Freed enjoys close scientific interactions with the other DRP PIs—Stephen Hughes, Alan Rein, Vinay Pathak, Wei-Shau Hu, Frank Maldarelli, Mary Kierney, and Alex Compton—and with DRP-associated investigators John Coffin and John Mellors.

Freed has long been an advocate for virology research and has worked to promote interactions among virologists. Since its launch in 2009, he has served as Editor-in-Chief of the open-access journal *Viruses*. He also serves as an Editor for *Journal of Molecular Biol*ogy and is on the editorial boards of several virology journals, including *Journal of Virol*ogy and *Retrovirology*. Starting in 2018, he is serving as an Associate Editor for Fields Virology. Freed has been an organizer for a number of international virology conferences, including the Cold Spring Harbor Retroviruses Meeting (2004); American Society for Cell Biology Conference on the Cell Biology of HIV-1 and Other Retroviruses (2006); the Keystone Meeting “Frontiers in HIV Pathogenesis, Therapy and Eradication” (2012); the Keystone Meeting “The Ins and Outs of Viral Infection: Entry, Assembly, Exit and Spread” (2014); two conferences sponsored by *Viruses*: “At the Forefront of Virus-Host Interactions (Basel, Switzerland, 2016) and “Breakthroughs in Viral Replication” (Barcelona, Spain, 2018); and the International Workshop of the Structure and Function of the gp41 Cytoplasmic Tail (National Cancer Institute, 2018). Freed is Co-Chair of the NIH Virology Interest Group and Co-Director of the University of Maryland Virology Program. In addition to ad hoc service on many national and international study sections and grant review panels, he was a member of the NIH AIDS Discovery and Development of Therapeutics (ADDT) Study Section from 2012 to 2017 and served as ADDT Chair from 2015 to 2017. The recognition of which Freed is perhaps most proud is an NCI Mentor of Merit Award for excellence in mentoring.

When not working, Freed enjoys running, hiking, skiing, biking, backpacking, fly fishing, and music, and spending time with his family.
